# Predictors of functional and morphological arterial wall properties in coronary artery disease patients with increased lipoprotein (a) levels before and after treatment with proprotein convertase subtilisin-kexin type 9 inhibitors

**DOI:** 10.1186/s12947-023-00313-9

**Published:** 2023-08-14

**Authors:** Andreja Rehberger Likozar, Miran Šebeštjen

**Affiliations:** 1 Department of Vascular Diseases, University Medical Centre Ljubljana, 1000 Ljubljana, Slovenia; 2 Faculty of Medicine, University of Ljubljana, Ljubljana, Slovenia; 3 Department of Cardiology, University Medical Centre Ljubljana, Zaloška 7, 1000 Ljubljana, Slovenia

**Keywords:** PCSK9 inhibitors, Lipoprotein (a), Inflammation, Hemostasis, Endothelial Function, Carotid-intima media thickness

## Abstract

**Background:**

In addition to proatherogenic properties, lipoprotein (a) (Lp(a)) has also pro-inflammatory, antifibrinolytic and prothrombogenic features. The aim of the current study was to identify the predictors of functional and morphological properties of the arterial wall in patients after myocardial infarction and increased Lp(a) levels at the beginning and after treatment with proprotein convertase subtilisin-kexin type 9 (PCSK9) inhibitors.

**Methods:**

Seventy-six post-myocardial infarction patients with high Lp(a) levels were included in the study. Ultrasound measurements of flow-mediated dilation of brachial artery (FMD), carotid intima-media thickness (c-IMT) and pulse wave velocity (PWV) were performed initially and after 6 months of treatment. At the same time points lipids, Lp(a), inflammatory and hemostasis markers were measured in blood samples.

**Results:**

In linear regression model FMD significantly correlated with age at first myocardial infarction (β = 0.689; *p* = 0.022), high-sensitivity C-reactive protein (β = -1.200; *p* = 0.009), vascular cell adhesion protein 1 (VCAM-1) (β = -0.992; *p* = 0.006), overall coagulation potential (β = 1.428; *p* = 0.014) and overall hemostasis potential (β = -1.473; *p* = 0.008). c-IMT significantly correlated with age at first myocardial infarction (β = 0.574; *p* = 0.033) and Lp(a) (β = 0.524; *p* = 0.040). PWV significantly correlated with systolic blood pressure (β = 0.332; *p* = 0.002), tumor necrosis factor alpha (β = 0.406; *p* = 0.002), interleukin-8 (β = -0.315; *p* = 0.015) and plasminogen activator inhibitor 1 (β = 0.229; *p* = 0.031). After treatment FMD reached statistical significance only in univariant analysis with systolic blood pressure (*r* = -0.286; *p* = 0.004) and VCAM-1 (*r* = -0.229; *p* = 0.024). PWV and c-IMT correlated with age (*r* = 0.334; *p* = 0.001 and *r* = 0.486; *p* < 0.0001, respectively) and systolic blood pressure (*r* = 0.556; *p* < 0.0001 and *r* = 0.233; *p* = 0.021, respectively).

**Conclusions:**

Our results suggest that age, systolic blood pressure, Lp(a) levels and other biochemical markers associated with Lp(a) are predictors of functional and morphological properties of the arterial vessel wall in post-myocardial patients with high Lp(a) levels initially. However, after 6 months of treatment with PCSK9 inhibitors only age and systolic blood pressure seem to be predictors of these properties.

**Trial registration:**

The protocol for this study was registered with clinicaltrials.gov on November, 3 2020 under registration number NCT04613167.

**Graphical Abstract:**

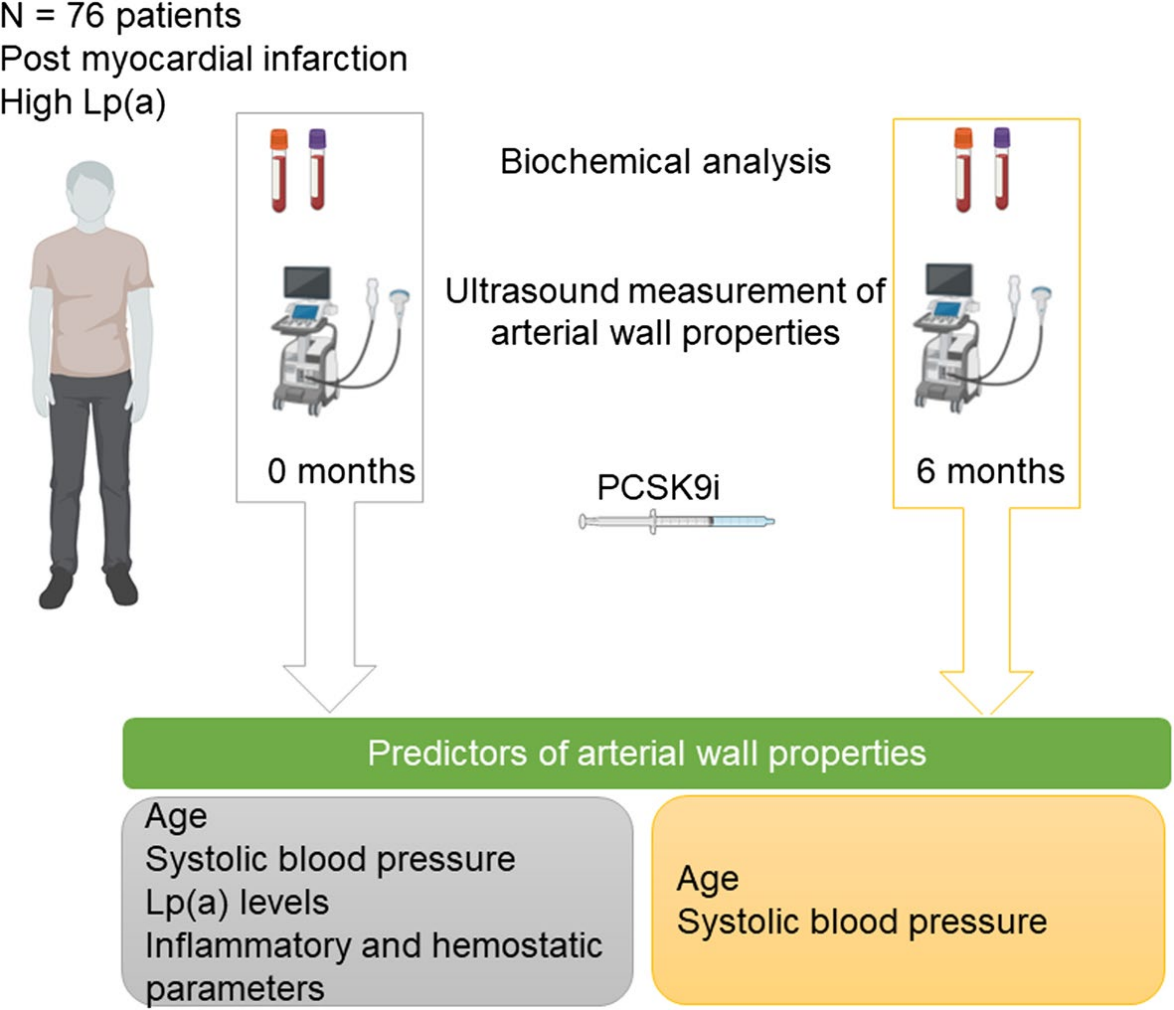

## Background

Despite the availability of the evidence based treatment with lipid-lowering drugs, target levels of low-density lipoprotein cholesterol (LDL-C) are achieved in less than 50% of the patients in secondary prevention [1] and LDL-C remains an important cardiovascular risk factor. Lipoprotein (a) (Lp(a)) is an additional cardiovascular risk factor resistant to current available lipid-lowering drugs, except for proprotein convertase subtilisin-kexin type 9 inhibitors (PCSK9i). Lp(a) consists of a LDL-like particle that is bound to apolipoprotein B100, which is then linked with apolipoprotein(a) (apo(a)) [2]. Lp(a) levels are genetically determined by the *LPA* gene, and they vary among individuals even by factor of 1000. However, within a single individual, Lp(a) levels are stable throughout life, with no influence of dietary habits or lifestyle [3]. Elevated levels of Lp(a) can occur in patients with otherwise normal lipid levels [4], while high Lp(a) levels in particular in patients with familiar hypercholesterolemia (FH) substantially increase their risk of cardiovascular events (CVE) [5]. It is therefore advisable for each person to have their Lp(a) levels determined at least once in a lifetime unless a specific treatment lowering Lp(a) is prescribed.

Proatherogenic properties of Lp(a) are due to similarity to LDL-C. However, entering of Lp(a) to the intima is not LDL receptor dependent, but it depends on Lp(a) plasma concentration, Lp(a) particle size and permeability of the endothelial cells. Lp(a) affinity for proteoglycans and fibronectin on the endothelial cells is higher than that of LDL-C [6]. Also, the atherogenic propensity of Lp(a) is stronger due to the fact that Lp(a) consist of both, LDL-C and apo(a) [7]. In addition to proatherogenic features Lp(a) has also pro-inflammatory, antifibrinolytic and prothrombogenic capabilities [8]. Pro-inflammatory properties of Lp(a) are associated with oxidized phospholipids that promote the formation of pro-inflammatory macrophages secreting the pro-inflammatory cytokines [9]. The key role in inflammation is played by cytokines, which are also produced by endothelial cells and smooth muscle cells as a response to various risk factors [10]. Pro-inflammatory cytokines are not involved only in the early phases of the atherosclerotic process, but also in the disruption of the atherosclerotic plaques via stimulating cell apoptosis, matrix deterioration and consequently the formation of thrombus [11]. Lp(a) participates in the process of atherothrombosis in several ways. First, it reduces platelet aggregation and activation [12]. Second, the procoagulant activity of Lp(a) is promoted by the increased expression of the tissue factor by endothelial cells [13], and consequently by the activation of extrinsic coagulation pathway leading to thrombus formation and intima fibrin deposition [14]. On the other hand, due to homology with plasminogen, Lp(a) can bind to plasminogen receptors and decrease the fibrinolytic potential [13].

Endothelial dysfunction is a first detectable functional change in the atherosclerotic process [15]. It is due to decreased bioavailability of nitric oxide found not only in patients with clinically evident atherosclerotic disease, but also in patients with risk factors [16]. Endothelial dysfunction was shown to be the independent predictor of future CVE in patients with coronary artery disease (CAD) and in patients at risk for CAD [17]. Lp(a) was shown to be associated with impaired endothelial function [18], and emerged as an independent predictor of future CVE [19]. Functional changes of the arterial vessel wall are followed by morphological changes which can be determined as an increased carotid intima media thickness (c-IMT) or increased pulse wave velocity (PWV) as a measure of the arterial stiffness. Contrary to brachial artery flow mediated dilation (FMD) Lp(a) was found not to be associated with c-IMT neither in FH children [20], nor in adults with FH already treated with statins [21]. However, PWV was independently associated with Lp(a) in patients with mild to moderate essential hypertension [22]. There is evidence that both, c-IMT [23] and PWV [24] predict future CVE.

In the current study we sought to identify the predictors of functional and morphological properties of the arterial wall measured as FMD, c-IMT and PWV. The predictors were searched among Lp(a) levels and biochemical markers directly or indirectly associated with Lp(a) in patients after myocardial infarction and significantly increased Lp(a) levels before and after treatment with PCSK9i.

## Patients, materials and methods

### Patients

**Fig. 1:**
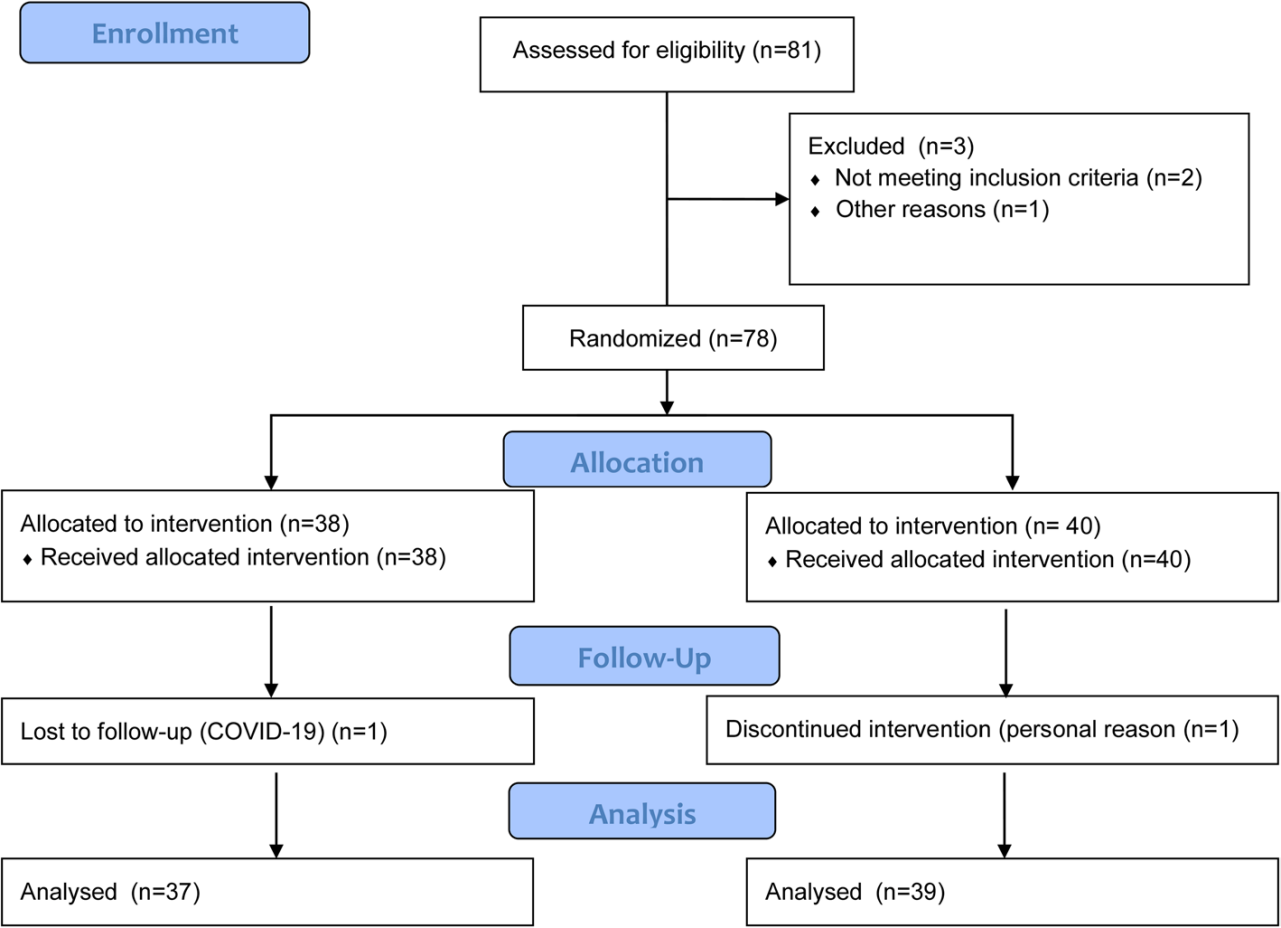
Flow diagram for patient inclusion. Initially, 81 patients with a stable CAD were enrolled in the study. Three patients were excluded in this phase, two of them did not meet the inclusion criteria, while one withdrew the informed consent before the start of the treatment. In total 78 patients were randomized to two groups, namely alirocumab or evolocumab treatment for 6 months. In each group, one patient was lost to follow up due to SARS-Cov2 infection and the other due to personal reasons. Finally, 76 patients were included in the analysis

Patient flow diagram is shown in Fig. 1. Patients who were at least 6 months after myocardial infarction and aged between 18 and 65 years with clinically stable CAD were enrolled in the study. In addition, patients eligible for inclusion in this study had serum Lp(a) levels of 1000 mg/l irrespective of LDL-C levels, or showed serum Lp(a) levels above 600 mg/l and LDL-C above 2.6 mmol/l. All patients were treated with β-blockers, antiplatelet drugs, angiotensin-converting enzyme (ACE) inhibitors, or angiotensin-receptor blockers and statins at the highest tolerated doses and ezetimibe as needed. There was no change in the therapy for at least 8 weeks prior to study entry. Initially, 81 patients were enrolled in the study. Three patients were excluded in this phase, two of them did not meet the inclusion criteria, while one withdrew the informed consent before the inclusion in the study.

Patients were treated with PCSK9i; alirocumab 150 mg subcutaneously or evolocumab 140 mg subcutaneously, every two weeks for 6 months. Given that there were no differences between the two treated groups, we combined them for the analysis.

Elevated liver transaminases more than three times the normal limit, serum creatinine above 200 mmol/l, or severe renal impairment and a history of acute illness in the past 6 weeks were the main exclusion criteria.

All the procedures performed in this study that involved human participants were carried out in accordance with the ethical guidelines of the 1964 Declaration of Helsinki. Approval for this study was obtained from the National Medical Ethics Committee of the Republic of Slovenia (reference number: KME 0120–357/2018/8). The study is registered with CinicalTrials under the number NCT04613167.

All patients signed a written informed consent prior to inclusion in the study.

### Clinical examination

The blood pressure was measured in the sitting position after a minimum of 10 min of rest. The average of three measurements was determined. Body mass index was calculated as weight in kilograms divided by square of height in meters, and anthropometric parameters were determined.

### Biochemical analysis

The blood for laboratory analyzes was taken in the morning after 12 h of fasting. Samples were collected from the antecubital vein into vacuum-sealed 5 ml tubes containing clot activator (Vacutube, LT Burnik, Slovenia). Serum was obtained by 15-min centrifugation at 2,000 × *g*. Total cholesterol, triglycerides, high-density lipoprotein cholesterol, apolipoproteins A1 and B were determined in the fresh serum by standard colorimetric or immunologic assays on an automated biochemistry analyzer (Fusion 5.1; Ortho-Clinical Diagnostics, USA). The same biochemistry analyzer was used to determine Lp(a) with the Denka reagent (Randox, UK), which contains apo(a) isoform-insensitive antibodies, and therefore showed minimal apo(a) size-related bias. The Friedewald formula [25] was used to calculate LDL-C. Thrombin activatable fibrinolysis inhibitor (TAFI) activity in plasma was measured using the chromogenic method utilizing Pefakit® TAFI reagents (Pentapharm, Switzerland) on an automated coagulation analyzer CS-2500 (Sysmex, Japan).

Plasminogen activator inhibitor-1 (PAI-1) antigen in plasma was measured using a classic sandwich ELISA (ASSERACHROM PAI-1, Diagnostica Stago, France). Tumor necrosis factor-alpha, high-sensitivity C-reactive protein (hs-CRP), interleukin 6, interleukin 8 and interleukin 10 levels in serum were measured using the Luminex’s xMAP® Technology utilizing magnetic beads coupled with specific antibodies, which allowed multiplexing. All analysis was performed according to manufacturer’s instructions (R&D Systems, UK). Overall coagulation potential (OCP), overall hemostasis potential (OHP) and overall fibrinolytic potential as supplementary parameters were determined as described before [26].

### Ultrasound measurements

Endothelial function was determined by FMD of the brachial artery, according to the guidelines [27] and as described in our previous study [28]. All procedures to measure the arterial stiffness were performed on the right common carotid artery as described before [28]. All of the measurements for c-IMT were conducted in accordance with the guidelines [29] and as described before [28].

### Statistical analysis

Kolmogorov–Smirnov test was used to determine the distribution of variables. Variables showing a normal distribution were expressed as means and standard deviation, while non-normally distributed variables were expressed as median and range. In correlation analysis of normally distributed variables Pearson's correlation coefficient was calculated, and Spearman's rank correlation coefficient was calculated for non-normally distributed variables. Multiple regression analysis was used to find the independent determinants for the variations in the properties of the arterial vessel wall. Statistical analysis was performed using IBM SPSS Statistics for Windows, (Version 25.0. Armonk, NY: IBM Corp.). *p* values < 0.05 were considered as statistically significant.

## Results

### Patient characteristics

A total of 76 patients with clinically stable CAD aged 50.9 ± 6.8 years at least 6 months after myocardial infarction, with mean age at the first coronary event of 43 years were included in the study. Their clinical and laboratory parameters before and after treatment are presented in Table 1. Most of the patients were males (86%), 11.4% were diabetics and 5.7% were active smokers. All patients were receiving statins at the highest tolerated dose without/with ezetimibe, except for two patients who were intolerant to statins, ACE inhibitors/angiotensin receptor blockers, β-blockers, and antiplatelet therapy.

**Table 1:** Patients clinical and biochemical characteristics before and after PCSK9i treatment

Parameter	Before	After
Body mass index (kg/m^2^)	28.5 ± 3.7	28.4 ± 3.9
Systolic blood pressure (mm Hg)	126 ± 14	124 ± 13
Diastolic blood pressure (mm Hg)	77 ± 8	79 ± 9
Total cholesterol (mmol/l)	4.26 ± 0.87	2.74 ± 0.95
HDL cholesterol (mmol/l)	1.17 ± 0.27	1.27 ± 0.32
LDL cholesterol (mmol/l)	2.33 ± 0.76	0.88 ± 0.82
Triglycerides (mmol/l)	1.68 ± 0.84	1.40 ± 0.87
Lipoprotein (a) (mg/l)	1444 (1199–1748)	1133 (820–1664)
Apolipoprotein B (g/l)	0.81 ± 0.22	0.46 ± 0.21
Apolipoprotein A1 (g/l)	1.34 ± 0.19	1.38 ± 0.19
hs-CRP (mg/l)	1.43 ± 1.44	1.69 ± 2.92
TNF-alpha (ng/l)	9.01 ± 14.30	9.92 ± 16.98
IL-6 (ng/l)	2.21 ± 2.08	2.19 ± 3.53
IL-8 (ng/l)	38.49 ± 156.25	29.34 ± 49.19
IL-10 (ng/l)	2.18 ± 9.57	1.52 ± 7.52
VCAM1 (mg/L)	0.72 ± 0.37	0.78 ± 0.41
TAFI (%)	99.18 ± 15.46	101.18 ± 15.40
PAI-1 antigen (ng/ml)	43.39 ± 29.26	54.49 ± 28.49
OCP (Abs-sum)	24.32 ± 5.37	25.21 ± 6.27
OFP (%)	71.54 ± 8.32	71.82 ± 8.21
OHP (Abs-sum)	6.89 ± 2.57	7.15 ± 2.94

Data are means ± standard deviation for data with normal distribution, and median (lower–upper quartiles) for data not distributed normally. *HDL cholesterol* high density lipoprotein cholesterol, *LDL cholesterol* low density lipoprotein cholesterol, *hs-CRP* high-sensitivity C reactive protein, *TNF-alpha* tumor necrosis factor alpha, *IL-6* interleukin 6, *IL-8* interleukin 8, *IL-10* interleukin 10, *VCAM1* vascular cell adhesion protein 1, *TAFI* thrombin activatable fibrinolysis inhibitor, *PAI-1* plasminogen activator inhibitor 1, *OCP* overall coagulation potential; *OFP* overall fibrinolytic potential, *OHP* overall hemostasis potential

### Correlation analyses

Before treatment with PCSK9i significant correlations for FMD were found with age (*r* = -0.305; *p* = 0.002), Lp(a) (*r* = -0.231; *p* = 0.020), hs-CRP (*r* = -0.305; *p* = 0.002), vascular cell adhesion protein 1 (VCAM1) (*r* = -0.225; *p* = 0.025), OCP (*r* = -0.234; *p* = 0.021) and OHP (*r* = -0.264; *p* = 0.009). No correlations were found between FMD and classical risk factor such as systolic or diastolic blood pressure and lipoproteins except Lp(a).

Functional properties measured as PWV correlated with age (*r* = -0.247; *p* = 0.013), systolic blood pressure (*r* = -0.2991; *p* = 0.003), Lp(a) (*r* = 0.208; *p* = 0.038) and PAI-1 (*r* = 0.218; *p* = 0.048). c-IMT as another measure of functional properties correlated with age (*r* = 0.488; *p* < 0.0001), systolic blood pressure (*r* = 0.207; *p* = 0.040), TAFI (*r* = 0.215; *p* = 0.036), PAI-1 (*r* = 0.256; *p* = 0.020) and OCP (*r* = 0.264; *p* = 0.009) (Table 2).

**Table 2:** Correlations of functional and morphological properties of the arterial wall with clinical and laboratory parameters before PCSK9i treatment

Parameter	FMD	PWV	c-IMT
Age (years)	***r* = -0.305; *p* = 0.002**	***r* = -0.247; *p* = 0.013**	***r* = -0.488; *p* < 0.0001**
Body mass index (kg/m^2^)	*r* = -0.059; *p* = 0.56	*r* = 0.104; *p* = 0.302	*r* = 0.161; *p* = 0.112
Systolic blood pressure (mm Hg)	*r* = -0.094; *p* = 0.35	***r* = -0.299; *p* = 0.003**	***r* = 0.207; *p* = 0.040**
Diastolic blood pressure (mm Hg)	*r* = -0.047; *p* = 0.64	*r* = 0.194; *p* = 0.053	*r* = 0.008; *p* = 0.937
Total cholesterol (mmol/l)	*r* = -0.021; *p* = 0.837	*r* = 0.076; *p* = 0.454	*r* = -0.029; *p* = 0.776
HDL cholesterol (mmol/l)	*r* = 0.014; *p* = 0.892	*r* = -0.009; *p* = 0.927	*r* = 0.106; *p* = 0.296
LDL cholesterol (mmol/l)	*r* = 0.046; *p* = 0.652	*r* = 0.081; *p* = 0.421	*r* = -0.102; *p* = 0.314
Triglycerides (mmol/l)	*r* = -0.153; *p* = 0.129	*r* = 0.018; *p* = 0.860	*r* = 0.066; *p* = 0.517
Lipoprotein (a) (mg/l)	***r* = -0.231; *p* = 0.020**	***r* = 0.208; *p* = 0.038**	*r* = -0.067; *p* = 0.510
Apolipoprotein B (g/l)	*r* = 0.003; *p* = 0.977	*r* = 0.062; *p* = 0.543	*r* = -0.043; *p* = 0.677
Apolipoprotein A1 (g/l)	*r* = 0.030; *p* = 0.772	*r* = -0.066; *p* = 0.517	*r* = 0.150; *p* = 0.144
hs-CRP (mg/l)	***r* = -0.263; *p* = 0.009**	*r* = 0.079; *p* = 0.439	*r* = -0.094; *p* = 0.358
TNF-alpha (ng/l)	*r* = 0.050; *p* = 0.625	*r* = 0.171; *p* = 0.091	*r* = -0.020; *p* = 0.843
Il-6 (ng/l)	*r* = -0.012; *p* = 0.907	*r* = -0.055; *p* = 0.591	*r* = -0.016; *p* = 0.876
IL-8 (ng/l)	*r* = 0.048; *p* = 0.636	*r* = -0.035; *p* = 0.735	*r* = -0.053; *p* = 0.605
IL-10 (ng/l)	*r* = 0.071; *p* = 0.486	*r* = -0.078; *p* = 0.440	*r* = 0-.025; *p* = 0.808
VCAM1 (mg/l)	***r* = -0.225; *p* = 0.025**	*r* = 0.042; *p* = 0.677	*r* = 0.037; *p* = 0.715
TAFI (%)	*r* = -0.060; *p* = 0.561	*r* = 0.109; *p* = 0.290	***r* = 0.215; *p* = 0.036**
PAI-1 (ng/ml)	*r* = 0.030; *p* = 0.129	***r* = 0.218; *p* = 0.048**	***r* = 0.256; *p* = 0.020**
OCP (Abs-sum)	***r* = -0.234 *p* = 0.021**	*r* = 0.057 *p* = 0.577	***r* = 0.264 *p* = 0.009**
OFP (%)	*r* = 0.119; *p* = 0.2 44	*r* = -0.054; *p* = 0.602	*r* = 0.004; *p* = 0.971
OHP (Abs-sum)	***r* = -0.264; *p* = 0.009**	*r* = 0.093; *p* = 0.367	*r* = 0.144; *p* = 0.161

*FMD* flow mediated dilation, *PWV* pulse wave velocity, *c-IMT* carotid intima media thickness, *HDL cholesterol* high density lipoprotein cholesterol, *LDL cholesterol* low density lipoprotein cholesterol, *hs-CRP* high-sensitivity C—reactive protein, *TNF-alpha* tumor necrosis factor alpha, *IL-6* interleukin 6, *IL-8* interleukin 8, *IL-10* interleukin 10, *VCAM1* vascular cell adhesion protein 1, *TAFI* thrombin activatable fibrinolysis inhibitor, *PAI-1* plasminogen activator inhibitor 1, *OCP* overall coagulation potential, *OFP* overall fibrinolytic potential, *OHP* overall hemostasis potential

After six months of treatment with PCSK9i FMD correlated with systolic blood pressure (*r* = -0.286; *p* = 0.004) and VCAM1 (*r* = -0.229; *p* = 0.024), while correlation with age was borderline significant (*r* = 0.319; *p* = 0.061).

Both morphological properties, PWV and c-IMT correlated with age (*r* = 0.334; *p* = 0.001 and *r* = 0.486; *p* < 0.0001 respectively) and systolic blood pressure (*r* = 0.556; *p* < 0.0001 and *r* = 0.233; *p* = 0.021 respectively). PWV also correlated with diastolic blood pressure (*r* = 0.386; *p* < 0.0001), while c-IMT correlated with TAFI (*r* = -0.263; *p* = 0.011) (Table 3).

**Table 3:** Correlations of functional and morphological properties of the arterial wall with clinical and laboratory parameters after PCSK9i treatment

Parameter	FMD	PWV	c-IMT
Age (years)	*r* = -0.3189; *p* = 0.061	***r* = -0.334; *p* = 0.001**	***r* = -0.486; *p* < 0.0001**
Body mass index (kg/m^2^)	*r* = -0.046; *p* = 0.435	*r* = 0.096; *p* = 0.365	*r* = 0.187; *p* = 0.142
Systolic blood pressure (mm Hg)	***r* = -0.286; *p* = 0.004**	***r* = 0.566; *p* < 0.0001**	***r* = 0.233; *p* = 0.021**
Diastolic blood pressure (mm Hg)	*r* = -0.093; *p* = 0.361	***r* = 0.386; *p* < 0.0001**	*r* = 0.097; *p* = 0.342
Total cholesterol (mmol/l)	*r* = -0.039; *p* = 0.701	*r* = -0.044; *p* = 0.454	*r* = -0.021; *p* = 0.834
HDL cholesterol (mmol/l)	*r* = 0.034; *p* = 0.738	*r* = 0.001; *p* = 0.996	*r* = 0.087; *p* = 0.339
LDL cholesterol (mmol/l)	*r* = 0-.036; *p* = 0.723	*r* = -0.096; *p* = 0.342	*r* = -0.01; *p* = 0.874
Triglycerides (mmol/l)	*r* = -0.071; *p* = 0.483	*r* = 0.061; *p* = 0.546	*r* = -0.013; *p* = 0.902
Lipoprotein (a) (mg/l)	*r* = 0.048; *p* = 0.638	*r* = 0.061; *p* = 0.550	*r* = -0.040; *p* = 0.697
Apolipoprotein B (g/l)	*r* = -0.060; *p* = 0.560	*r* = -0.066; *p* = 0.522	*r* = -0.039; *p* = 0.707
Apolipoprotein A1 (g/l)	*r* = 0.039; *p* = 0.705	*r* = -0.014; *p* = 0.895	*r* = 0.077; *p* = 0.454
hs-CRP (mg/l)	*r* = -0.107; *p* = 0.295	*r* = 0.145; *p* = 0.153	*r* = -0.038; *p* = 0.715
TNF-alpha (ng/l)	*r* = -0.035; *p* = 0.733	*r* = 0.096; *p* = 0.350	*r* = -0.029; *p* = 0.777
IL-6 (ng/l)	*r* = -0.124; *p* = 0.164	*r* = 0.062; *p* = 0.548	*r* = 0.109; *p* = 0.288
IL-8 (ng/l)	*r* = 0.131; *p* = 0.200	*r* = 0.058; *p* = 0.527	*r* = -0.015; *p* = 0.881
IL-10 (ng/l)	*r* = 0.026; *p* = 0.803	*r* = -0.045; *p* = 0.664	*r* = 0.152; *p* = 0.138
VCAM1 (mg/l)	***r* = -0.229; *p* = 0.024**	*r* = -0.060; *p* = 0.559	*r* = -0.043; *p* = 0.677
TAFI (%)	*r* = 0.136; *p* = 0.194	*r* = 0.148; *p* = 0.157	***r* = -0.263; *p* = 0.011**
PAI-1 (ng/ml)	*r* = -0.173; *p* = 0.122	*r* = 0.055; *p* = 0.627	*r* = 0.164; *p* = 0.146
OCP (Abs-sum)	*r* = -0.107 *p* = 0.296	*r* = 0.147 *p* = 0.150	*r* = 0.118 *p* = 0.251
OFP	*r* = -0.014 *p* = 0.893	*r* = -0.122; *p* = 0.235	*r* = -0.009; *p* = 0.929
OHP	*r* = -0.041; *p* = 0.687	*r* = 0.172; *p* = 0.091	*r* = 0.091; *p* = 0.378

*FMD* flow mediated dilation, *PWV* pulse wave velocity, *c-IMT* carotid intima media thickness, *HDL cholesterol* high density lipoprotein cholesterol, *LDL cholesterol* low density lipoprotein cholesterol, *hs-CRP* high-sensitivity C reactive protein, *TNF-alpha* tumor necrosis factor alpha, *IL-6* interleukin 6, *IL-8* interleukin 8, *IL-10* interleukin 10, *VCAM1* vascular cell adhesion protein 1, *TAFI* thrombin activatable fibrinolysis inhibitor, *PAI-1* plasminogen activator inhibitor 1, *OCP* overall coagulation potential, *OFP* overall fibrinolytic potential, *OHP* overall hemostasis potential

### Multivariant analyses

The importance of variables that showed significance in the univariate analysis in predicting FMD before treatment with PCSK9i was tested in several linear regression models. Table 4 presents the conclusions of the best model which explained 98.8% of FMD variability (*p* = 0.020).

**Table 4:** Linear regression model of predictors of flow-mediated dilatation of the brachial artery

Parameter	ß	Tolerance	p
Age at first MACE (years)	**0.689**	**4.394**	***p* = 0.022**
Systolic blood pressure (mm Hg)	-0.401	-3.008	*p* = 0.057
Lipoprotein (a) (mg/l)	-0.248	-2.074	*p* = 0.130
hs-CRP (mg/l)	**-1.200**	**-5.994**	***p* = 0.009**
VCAM1 (mg/l)	-0.992	**-7.169**	***p* = 0.006**
OCP (Abs-sum)	**1.428**	**5.196**	***p* = 0.014**
OHP (Abs-sum)	**-1.473**	**-6.248**	***p* = 0.008**

R^2^ (part of variability explained with the model) = 0.988, *p* = 0.020, *MACE* major adverse cardiac events, *hs-CRP* high-sensitivity C-reactive protein, *VCAM1* vascular cell adhesion protein 1, *OCP* overall coagulation potential, *OHP* overall hemostasis potential

Variables such as age at the first acute coronary event, hs-CRP, VCAM1, OCP and OHP reached statistical significance in this model. In linear regression model that explained 52.7% (*p* = 0.001) variability of PWV (Table 5), systolic blood pressure, tumor necrosis factor-alpha, interleukin 8 and PAI-1 emerged as statistically important predictors of PWV.

**Table 5:** Linear regression model of predictors of pulse wave velocity in the carotid artery

Parameter	ß	Tolerance	p
Systolic blood pressure (mm Hg)	**0.332**	**3.164**	***p* = 0.002**
Lipoprotein (a) (mg/l)	0.125	1.214	*p* = 0.229
Apo A1 (g/l)	-0.129	-1.284	*p* = 0.203
TNF-alpha (ng/l)	**0.406**	**3.229**	***p* = 0.002**
IL-8 (ng/l)	**-0.315**	**-2.497**	***p* = 0.015**
PAI-1 (ng/ml)	**0.229**	**2.194**	***p* = 0.031**

R^2^ (part of variability explained with the model) = 0.527, *p* = 0.001, *Apo A1* apolipoprotein A1, *TNF-alpha* tumor necrosis factor alpha, *IL-8* interleukin 8, PAI-1 – plasminogen activator inhibitor 1

Age at the first acute coronary event, Lp(a), interleukin 8, VCAM1, OCP and OHP (Table 6) reached statistical significance in the linear regression model that explained 97.5% (*p* = 0.044) of variability of c-IMT. After 6 months of treatment with PCSK9i only three parameters at most reached statistical significance in univariant analysis for FMD, PWV and c-IMT, hence linear regression analysis was not possible.

**Table 6:** Linear regression model of predictors of carotid intima media-thickness

Parameter	ß	Tolerance	p
Age at first MACE (years)	**0.574**	**3.753**	***p* = 0.033**
Systolic blood pressure (mm Hg)	0.330	2.160	*p* = 0.120
Lipoprotein (a) (mg/l)	**0.524**	**3.470**	***p* = 0.040**
IL-8 (ng/l)	0.539	2.684	*p* = 0.075
PAI-1 (ng/ml)	-0.616	-2.647	*p* = 0.077
OCP (Abs-sum)	0.388	2.334	*p* = 0.102

R^2^ (part of variability explained with the model) = 0.975, *p* = 0.044, *MACE* major adverse cardiac events, *IL-8* interleukin 8, *PAI-1* plasminogen activator inhibitor 1, *OCP* overall coagulation potential

## Discussion

To the best of our knowledge, our study is the first to examine the influence of both, classical risk factors, as well as inflammatory risk factors and factors related to disorders in coagulation fibrinolytic process on the functional and morphological properties of the arterial vessel wall in patients with CAD before and after treatment with PCSK9i. The uniqueness of our research is in the selection of the patients, as we have included the patients in the stable phase of CAD, with well-controlled risk factors other than lipids. Despite treatment with maximum tolerated doses of statins and ezetimibe if needed, their LDL-C levels did not reach the goal values as suggested by the guidelines [30]. In addition, all patients included in our study had significantly increased Lp(a) values. PCSK9i are the first drugs that lower both, LDL-C and Lp(a) levels [31, 32]. Moreover, PCSK9i also decrease the occurrence of CVE [31, 32]. In the HUYGENS study in post-myocardial infarction patients with similar values of total and LDL-C and significantly lower Lp(a) values, treatment with evolocumab significantly improved the morphological characteristics of the atherosclerotic lesions by reducing the volume of the lipid core and increasing the thickness of the fibrous cap [33].

Not surprisingly both, functional and morphological properties of the arterial vessel wall before and after treatment with PCSK9i, were statistically significantly associated with age in our study. Considering that elevated concentrations of LDL-C and Lp(a) levels in particular are genetically determined [34], our patients most likely had elevated levels of both since their early age. The total burden of a single risk factor is composed of the magnitude of the single risk factor and the length of the exposure [35]. All patients in our study were treated with statins, which effectively lower LDL-C and also improve the functional and morphological properties of the arterial vessel wall [36]. Statins also increase Lp(a) levels regardless of the type, dose, or duration of the treatment [37]. However, we have no data on the potential negative impact of the statin treatment on arterial wall parameters. In any case, the overall effects of the statin treatment on the arterial wall properties and the occurrence of acute CVE are positive. This also explains that neither functional nor morphological characteristics were associated with LDL-C concentration in our study. On the other hand, both, functional and morphological properties of the arterial vessel wall were related to the Lp(a) levels. In patients without clinically evident atherosclerosis neither FMD nor c-IMT were associated with Lp(a) levels, which were significantly lower compared to our patients [38]. The LDL-C concentration, which was only moderately increased (mean 3.13 mmol/l), was associated with both, FMD and c-IMT. In the Atherosclerosis Risk in Communities (ARIC) Study, which included 15,124 patients without clinically evident atherosclerosis, Lp(a) proved to be weak but statistically significant predictor of increased c-IMT [39]. Similar to our study, Bos et al. did not find any direct relationship between c-IMT and Lp(a) values [21]. In 191 patients with FH and very diverse Lp(a) values ​​with or without clinically evident atherosclerosis, no differences were found in c-IMT and the incidence of the atherosclerotic plaques in the carotid arteries between the groups with Lp(a) values ​​above or below 300 mg/dl. The main difference between our and the latter study is that all our patients had Lp(a) values ​​above 600 mg/dl. However, in both studies the patients were treated with the maximum tolerated dose of statin and, if necessary, also with ezetimibe. In contrast, c-IMT and PWV were not significantly higher in children with heterozygous FH and moderately elevated Lp(a) values than in healthy peers, but FMD was significantly reduced [20]. This can be explained by both, the significantly increased concentration of LDL-C and only moderately increased values of Lp(a), and the fact that functional changes appear much earlier than the morphological ones. In patients with an average age of 12 years, the duration of the risk factors is definitely an important factor. The role of the increased values of Lp(a) for endothelial dysfunction is also confirmed by a study performed in 11-year-old children with endothelial dysfunction. These children had only moderately increased concentration of LDL-C and highly increased values ​​of Lp(a) [18]. Lp(a) is involved in the process of atherosclerosis due to its structural similarity to LDL-C. However, in patients with elevated LDL-C as well as Lp(a), it is difficult to distinguish which factor is a major contributor.

Moreover, the second main part of Lp(a), apo(a), is structurally very similar to plasminogen, which is one of the most important components of the coagulation fibrinolytic system [8]. Since Lp(a) has structural homology to plasminogen, it can bind to plasminogen receptors on the surface of platelets. In this way Lp(a) can prevent the interaction between plasminogen and tissue plasminogen activator decreasing fibrinolytic capacity and thus increasing the coagulation side of the hemostatic balance. The disturbed balance of the hemostatic potential in favor of coagulation participates in all phases of the atherosclerotic process, from the endothelial dysfunction to the morphological changes, and finally to an acute CVE [13]. In the coagulation fibrinolytic system there are many factors that depend on each other. By measuring the individual factors such as PAI-1 and TAFI, we cannot sufficiently assess the overall state of the coagulation fibrinolytic system. Therefore, in addition to individual factors in the coagulation fibrinolytic system, we have also measured the OHP. OHP can distinguish between the hypocoagulability due to low activity of the coagulation factors, and the hypercoagulability, which also includes ischemic heart disease [26]. To the best of our knowledge no previous study investigated the relationship between the functional and/or morphological properties of the arterial vessel wall and OHP, overall fibrinolytic potential and OCP as additional parameters. In our study, both, PAI-1 antigen and TAFI proved to be the predictive factors for the morphological, but not the functional properties of the arterial vessel wall. In the ARIC study, a statistically significant association of the PAI-1 antigen with c-IMT was determined in 457 patients without significant atherosclerotic disease [40]. Another coagulation factor that was associated with c-IMT in our study is TAFI. Similar to PAI-1, TAFI is one of the most potent inhibitors of fibrinolysis [41]. In patients with coronary atherosclerosis, defined as stenosis of the coronary artery greater than 20%, the concentration of TAFI in venous samples was higher than in patients without coronary atherosclerosis. However, the concentration of TAFI in intracoronary samples was significantly higher compared to venous samples in both groups. In the group of patients with coronary atherosclerosis, the concentration of TAFI was significantly higher in those who had an acute coronary event in the past or required surgical or percutaneous revascularization [42]. In patients in the early phase of an acute cerebrovascular event, decreased fibrinolytic activity was associated with the increased levels of both, TAFI and PAI-1 [43], whereas in patients with type II diabetes, TAFI was associated with the decreased fibrinolytic activity independent of PAI-1 [44].

To the best of our knowledge, there is no data in the literature on the effects of OHP, OCP or overall fibrinolytic potential on the functional and/or morphological properties of the arterial vessel wall. In our study, OCP and OHP proved to be independent predictive factors for both, functional (FMD) and morphological (c-IMT) properties of the arterial vessel wall. Guven et al. showed that FMD is not associated with TAFI in a group of 35 patients with hypercholesterolemia without obvious atherosclerotic disease. However, they also showed that in those patients treated with simvastatin (40 mg daily) FMD significantly improved, and also the TAFI levels significantly reduced, but without their mutual association [45]. TAFI plays an important role in the various critical interactions between the endothelial function, coagulation, and fibrinolysis. Because of its role in the fibrinolytic system, TAFI may be implicated in the atherothrombotic diseases, after plaque rupture in consequent acute CVE. Of course, the results of Guven et al. cannot be compared with our results, since they included the patients with a risk factor, without prior CAD or even conditions after an acute coronary event [45], while all our patients survived an acute coronary event. Hence, the TAFI values were significantly different between their and our groups [45]. No association between c-IMT and PAI-1 was found in apparently healthy subjects without prior therapy [46]. In patients with combined hyperlipidemia, without the presence of other risk factors, the concentration of total TAFI proved to be an independent predictive factor for c-IMT. However, the association with FMD was statistically significant only in univariate analysis and no association was found with PAI-1 [47]. All the aforementioned factors participate in one way or another in the process of fibrinolysis, but their connections with the functional and morphological properties of the arterial vessel wall are not unambiguous. The factors involved in the coagulation process are also related to the functional and morphological properties of the arterial vessel wall, but here the results are also contradictory [48, 49].

## Conclusions

In the current study, we determined which risk factors predict the functional and morphological characteristics of the arterial vessel wall before and after treatment with PCSK9i in post-myocardial infarction patients with moderately increased LDL-C and strongly increased Lp(a) levels and well-controlled other risk factors. In addition to age and arterial blood pressure both, the functional and the morphological properties of the arterial vessel wall of these patients, depend on the values of Lp(a) and related factors of inflammation and hemostasis before PCSK9i treatment. However, after PCSK9i treatment, only well-recognized risk factors such as age and blood pressure seem to predict the arterial wall properties. Given that post-treatment LDL-C levels were ideally controlled, while Lp(a) levels were still significantly elevated, it will be interesting to determine whether the additional distinct lowering of Lp(a) with Lp(a)-specific drugs changes these relationships.

## Data Availability

Raw data were generated at University Medical Centre Ljubljana. Derived data supporting the findings of this study are available from the corresponding author on request.

## Abbreviations

CAD: Coronary artery disease
c-IMT: Carotid intima-media thickness
CVE: Cardiovascular events
FMD: Flow-mediated dilation of brachial artery
HDL: High density lipoprotein cholesterol
hs-CRP: High-sensitivity C reactive protein
IL-6,8,10: Interleukin 6,8,10
LDL-C: Low-density lipoprotein cholesterol
Lp(a): Lipoprotein (a)
MACE: Major adverse cardiac events
OCP: Overall coagulation potential
OFP: Overall fibrinolytic potential
OHP: Overall hemostasis potential
PAI-1: Plasminogen activator inhibitor 1
PCSK9i: Proprotein convertase subtilisin-kexin type 9 inhibitors
PWV: Pulse wave velocity
VCAM1: Vascular cell adhesion protein 1
TAFI: Thrombin activatable fibrinolysis inhibitor
TNF-alpha: Tumor necrosis factor alpha
